# Horner’s syndrome after an ultrasound-guided fine-needle aspiration puncture of a thyroid nodule

**DOI:** 10.1530/EDM-25-0016

**Published:** 2025-04-07

**Authors:** Alexandra Abegão Matias, Teresa Sabino, José Silva-Nunes

**Affiliations:** ^1^ Department of Endocrinology, Diabetes and Metabolism, Unidade Local de Saúde São José, Curry Cabral Hospital, Lisbon, Portugal; ^2^ NOVA Medical School/ Faculdade de Ciências Médicas, New University of Lisbon, Lisbon, Portugal; ^3^ Centro Clínico Académico de Lisboa, Lisbon, Portugal; ^4^ Health and Technology Research Center (H&TRC), Escola Superior de Tecnologia da Saúde de Lisboa, Lisbon, Portugal

**Keywords:** Horner’s syndrome, thyroid, fine needle aspiration puncture

## Abstract

**Summary:**

Horner’s syndrome is a rare condition that results from damage to the oculosympathetic chain. The classical presentation consists of miosis, unilateral ptosis and hemifacial anhidrosis due to a deficiency of sympathetic activity. Although it has been described as a result of different types of trauma, we present the first clinical report of Horner’s syndrome that was developed after a fine-needle aspiration puncture of a thyroid nodule. A 48-year-old woman with a non-toxic multinodular goiter underwent an ultrasound-guided fine-needle aspiration for the second time for a nodule located at the right thyroid lobe. Four hours after the procedure, she developed homolateral eyelid ptosis, miosis and enophthalmos and went to the emergency department for observation. Structural causes potentially related to the manifestations were excluded. Horner’s syndrome was diagnosed and the patient was discharged with symptomatic measures. Three months after the event, the patient reported partial improvement. We discuss the pathophysiology associated with Horner’s syndrome, the association with thyroid diagnostic and therapeutic procedures, clinical presentation, patient management and prognosis. Although fine-needle aspiration of a thyroid nodule has few associated complications, Horner’s syndrome should be considered when the patient develops ophthalmologic symptoms. Preventive measures should be observed to minimize its occurrence.

**Learning points:**

- Horner’s syndrome is a potential complication of diagnostic and therapeutic procedures directed at the thyroid gland, including ultrasound-guided fine-needle aspiration.
- It is characterized by eyelid ptosis, miosis, enophthalmos and anhidrosis homolateral to the lesion.
- The diagnosis of Horner’s syndrome is clinical after excluding structural causes and establishing the temporal relationship between the procedure and the manifestations.
- There is no targeted treatment for Horner’s syndrome, but it can be prevented.

## Background

Horner’s syndrome is a rare condition, with a cumulative 10-year incidence in the adult population of 2.95 per 100,000 people (1). This disease corresponds to a set of manifestations secondary to a lesion of the oculosympathetic chain. This pathway consists in three sets of neurons that originates from the hypothalamus and runs to the eye. The vast majority (>80%) of lesions affect the second-order or the third-order neurons. Typically, Horner’s syndrome presents with palpebral ptosis, miosis, enophthalmos and facial anhidrosis ipsilateral to the side of the neurological lesion (1). It has been reported as a consequence of compressive effect, with a starting point in the thyroid gland, even of benign etiology (2, 3). It has also been described as a rare complication of invasive procedures related to the thyroid gland, namely after thyroidectomy with or without lymphadenectomy (4). More recently, it has been described during thyroid nodules ablative therapies (5, 6). In 2023, a clinical case of a patient with suspected nodal metastasis of a papillary thyroid carcinoma who developed Horner’s syndrome after cervical lymph node fine-needle aspiration was published (7).

Fine-needle aspiration is the recommended diagnostic procedure for cytological evaluation of thyroid nodules. When this procedure is ultrasound-guided, we can achieve greater accuracy while minimizing the risk of side effects. Usually, associated complications are rare, of minimal severity and transient, such as mild pain (up to 8.9%), hemorrhage (0.3–2.3%) and metastatic dissemination of malignant thyroid disease (8). To the best of our knowledge, we are reporting the first patient with Horner’s syndrome as a complication of ultrasound-guided fine-needle aspiration puncture of a thyroid nodule.

## Case presentation

**Figure 1:**
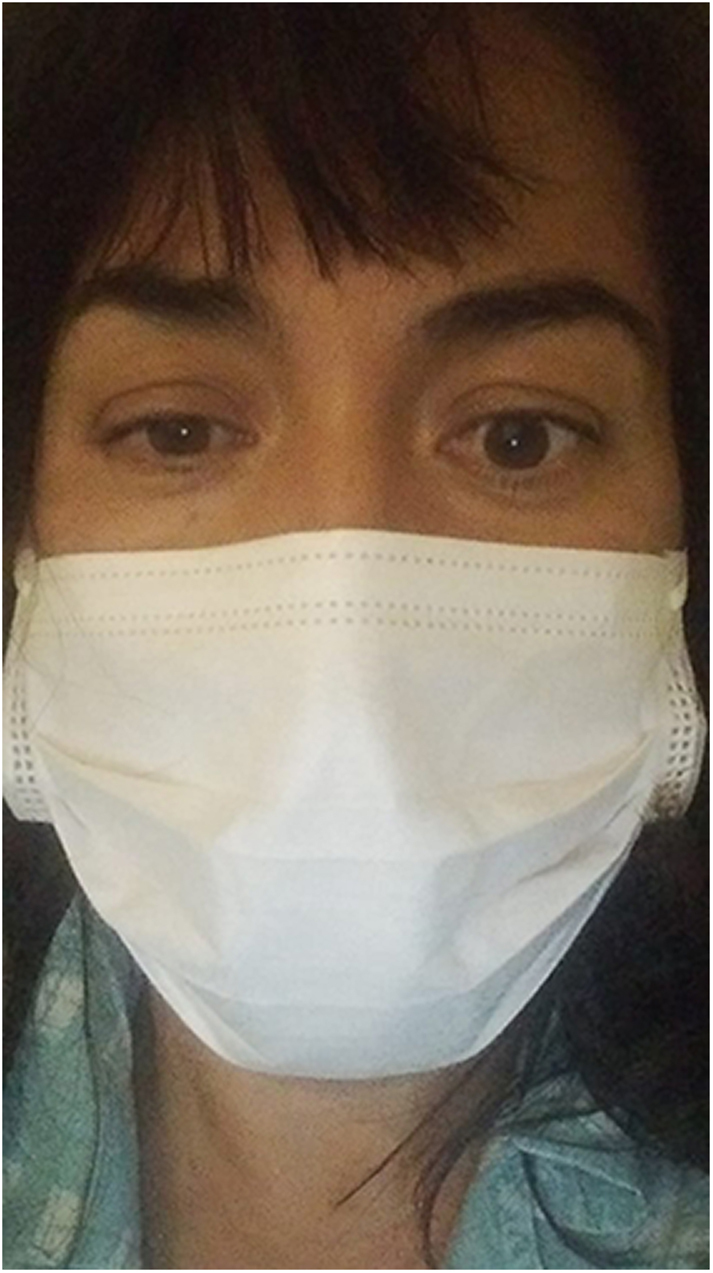
Anisocoria, miosis and partial ptosis of the right eye in a 48-year-old female patient after ultrasound-guided fine-needle aspiration puncture of a thyroid nodule located at the right lobe.

A 48-year-old Caucasian female patient was referred to our department because she was diagnosed with non-toxic multinodular goiter. Concomitantly, she presented class I obesity (body mass index of 32 kg/m^2^) and high blood pressure. The patient reported cervical discomfort and sporadic dysphonia, without associated dyspnea or dysphagia. She has no personal history of radiation exposure or family history of malignant thyroid disease. On cervical palpation, no thyroid nodule or locoregional adenomegalies were identified. Thyroid ultrasound detected several infracentimeter nodules, with the largest located in the upper third of the right lobe, near the thyroid capsule, isoechogenic, well delimited and measuring 11 × 13 × 17 mm (transverse × anteroposterior × longitudinal diameters). Analytically, she was in a state of euthyroidism, with negative anti-thyroglobulin and anti-thyroid peroxidase antibodies. She underwent a first ultrasound-guided fine-needle aspiration puncture of the referred nodule, without complications. The cytological result was ‘non-diagnostic’ according to the Bethesda category. Consequently, a second fine-needle aspiration puncture was performed 4 months later. During this procedure, a 23-gauge needle was used for two ultrasound-guided fine-needle aspiration punctures of the same nodule, without immediate symptoms. Four hours after the procedure, the patient reported the progressive onset of ocular discomfort in the right eye, blurred vision and ipsilateral eyelid ptosis, without accompanying inflammatory signs. The patient denied any kind of local trauma; no changes in eye movements or in visual acuity were present. She reported progressive worsening with miosis and ptosis of the right eye more evident about 24 h after puncture. She was admitted to the emergency room with anisocoria, with a decrease in the diameter of the right pupil, partial ptosis and enophthalmos of the right eye (Fig. 1). Pupillary reflexes and eye movements were preserved. The patient had no other cranial nerve abnormalities.

## Investigation

The patient underwent computed tomography of the brain and supraaortic vessels; no vascular dissections or other structural lesions were detected. The patient remained for 24 h in the emergency department for monitoring.

## Treatment

Horner’s syndrome secondary to fine-needle aspiration of the right thyroid nodule was assumed, and she was discharged with symptomatic measures. The patient was instructed to perform frequent eye lubrication, and no other targeted therapy was recommended.

## Outcome and follow-up

**Figure 2:**
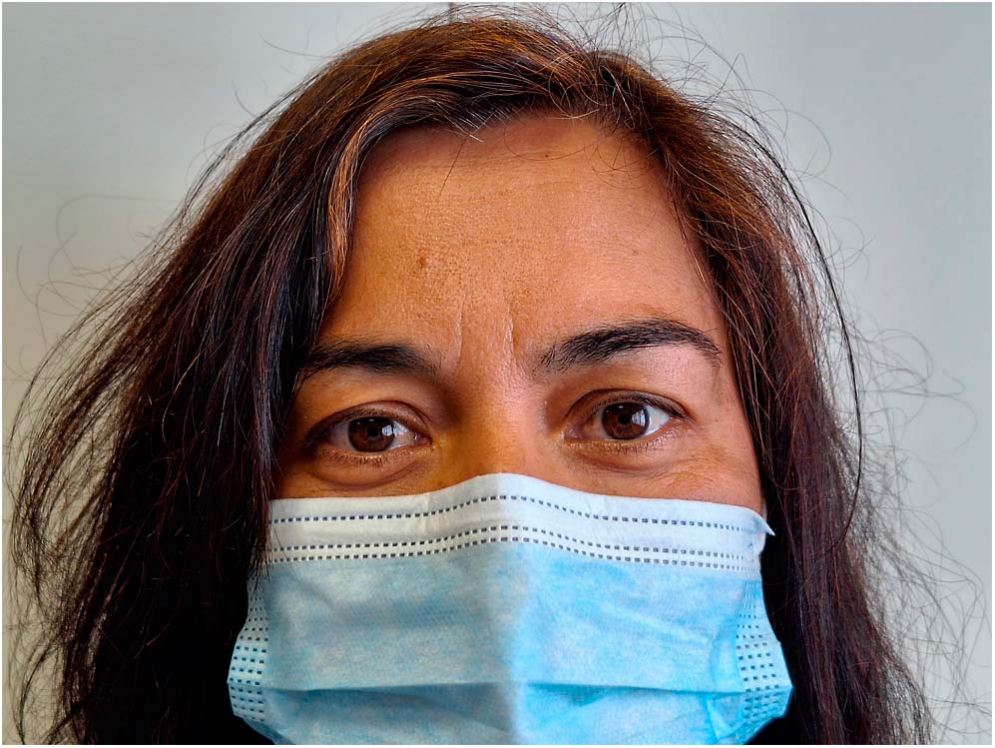
Appearance of the patient after resolution of Horner’s syndrome.

Three months after the procedure, the patient reported a progressive global improvement in symptoms, but she still maintained evening eyelid ptosis. Two years after the procedure, the patient reports complete resolution of Horner’s syndrome (Fig. 2).

## Discussion

This clinical case reports a 48-year-old woman who developed Horner’s syndrome some hours after being submitted to an ultrasound-guided fine-needle aspiration of a thyroid nodule. After excluding structural causes potentially responsible for the syndrome, and considering the temporal relationship between the procedure and the subsequent onset of manifestations, it was assumed to be consequence of the fine-needle aspiration of the thyroid nodule. This is the first report that associates Horner’s syndrome with fine-needle aspiration of thyroid nodules.

Horner’s syndrome results from the interruption of the oculosympathetic chain (1). The oculosympathetic chain is made up of first-order, second-order and third-order neurons. First-order neurons are in the posterolateral hypothalamus, extending along the lateral brainstem (1, 9). Synapsis with second-order neurons occurs at the ciliospinal center of Budge and Waller, located in the intermediate-lateral gray column of the spinal cord at the level of C8–T1 (9). In turn, preganglionic sympathetic fibers emerge from the ciliospinal center of Budge and Waller, pass near to the pulmonary apex, ascend through the carotid sheath and synapse with third-order neurons located in the superior cervical sympathetic ganglion (9). Postganglionic sympathetic fibers extend along the vascular path of the internal carotid artery. Upon reaching the cavernous sinus, they are conducted parallel to the VI cranial nerve until they reach the ophthalmic division of the V cranial nerve, running to the orbit (9). There, they are responsible for the innervation of the pupillary sphincter and accessory muscles responsible for eyelid retraction.

The manifestations and etiology of Horner’s syndrome will depend on the level at which the lesion occurs. Lesions starting from the thyroid gland may affect pre- or post-ganglionic sympathetic nerve fibers, affecting both second- and third-order neurons. Most lesions occur at the level of preganglionic sympathetic fibers (9). In pediatric and adult populations, the main identified cause of Horner’s syndrome is the surgical procedure, especially cervical surgery (1).

Horner’s syndrome has also been described as a rare iatrogenic complication of several procedures directed to the thyroid gland, from thyroid surgery to minimally invasive procedures on thyroid nodules. A recent review, which gathered the reported cases of Horner’s syndrome related to thyroid surgery, showed that the two main symptoms described were miosis and ptosis of the upper eyelid (1). Several cases of Horner’s syndrome after video-assisted minimally invasive thyroidectomy have been described as a complication of this procedure, even without other associated complications such as hemorrhage, vocal cord paresis or iatrogenic hypoparathyroidism (1, 3). The incidence of Horner’s syndrome after thyroidectomy has been reported to be less than 1% (1). The onset of manifestations is variable, ranging from immediately after the procedure to 72 h after (1). In our clinical report, the patient’s main complaints included miosis and eyelid ptosis. The immediate onset of complaints was followed by progressive worsening, which may explain the temporal variability described in previous reports.

Horner’s syndrome can result from the compressive action of an enlarged thyroid gland or from iatrogenesis induced during diagnostic or treatment procedures. To the best of our knowledge, this is the first report of Horner’s syndrome associated with ultrasound-guided fine-needle aspiration puncture of a thyroid nodule, a very common procedure, demonstrating how unusual this complication is. However, the probability of this happening is explained by the close anatomical relationship that exists between the cervical sympathetic trunk and the thyroid gland. Usually, cervical sympathetic trunk is located posteriorly and internally to the carotid sheath; however, anatomical variations may occur facilitating the occurrence of injuries to this structure (6). Multiple explanations have been advanced regarding the pathophysiology of Horner’s syndrome associated with manipulation of the thyroid gland, although there is no well-defined etiology. One of the possibilities is direct injury to the oculosympathetic chain, whether mechanical or thermal. Sympathetic ganglion may present ultrasound characteristics similar to other cervical structures, such as lymph nodes (7). Furthermore, the cervical sympathetic trunk has a connection with other adjacent nervous structures and its own vascularization; injuries to these structures may compromise the oculosympathetic chain (6). Nerve branches between the recurrent laryngeal nerve and the cervical sympathetic trunk have been described, which is why manipulation of the recurrent laryngeal nerve may induce Horner’s syndrome. In addition, the sympathetic trunk is supplied by a branch of the inferior thyroid artery. Therefore, its manipulation and injury can, consequently, lead to cervical sympathetic trunk ischemia and Horner’s syndrome. The occurrence of hematomas or edema secondary to inflammation induced by local procedures may cause compression of the cervical sympathetic trunk, albeit transiently. In this case, as the bruises will likely be reabsorbed and the inflammation will regress over time, Horner’s syndrome will also remit. It is not always possible to clarify the exact mechanism that has triggered Horner’s syndrome, as in our clinical case report.

In order to minimize the occurrence of this complication, an adequate pre-procedure assessment of the structures adjacent to the thyroid gland must be performed. The middle cervical sympathetic ganglion is located at the inferior level of the thyroid gland and lateral to the common carotid artery, which is visible in 41% of ultrasound images (10). A sympathetic ganglion can resemble a lymph node: can be round or oval, less than 1 centimeter in short axis and with strong echo signals in its outer layer and dotted echo signals inside (7). The correct identification of this structure and its distinction from ganglionic, muscular structures or thyroid nodules may reduce the risk for lesion. Procedure-induced pain may limit the patient’s ability to remain immobile, increasing the risk of injury to the sympathetic trunk structures. The use of local analgesia or sedation for hypersensitive patients reduces that risk, especially regarding ablative therapies (5).

Horner’s syndrome does not require specific treatment. Some of the reported clinical cases refer to self-limited administration of methylcobalamin and intravenous or oral corticosteroid therapy (6). It has been proposed that these drugs promotes edema reduction related to neuronal injury and contributes to neuronal repair (6). However, even after starting this therapy, manifestations may last for months until complete resolution. Less often, Horner’s syndrome may not be completely resolved, and complaints remain as permanent sequelae (1). In this clinical report, the patient did not undergo any treatment and she reported a progressive global improvement in symptoms. Although the patient still reports minor symptomatology 3 months after the procedure, there was an improvement, which reinforces the transient nature of this syndrome. Eventually, it can be anticipated that the use of corticosteroid therapy could bring some benefits, namely a faster improvement of complaints. However, in the case previous reported in the literature in which methylcobalamin was given, 5 months after the procedure, the patient still had miosis and ptosis (6).

Although it is a rare and non-life-threatening condition, risk for Horner’s syndrome should be considered during diagnostic or therapeutic interventions involving the thyroid gland. This is the first report that associates Horner’s syndrome with fine-needle aspiration of thyroid nodules. As Horner’s syndrome significantly impacts on the patient’s physical appearance and, consequently, on the quality of life, all procedures must be considered in order to minimize the risk of its occurrence.

## Declaration of interest

The authors declare that there is no conflict of interest that could be perceived as prejudicing the impartiality of the work reported.

## Funding

This research did not receive any specific grant from any funding agency in the public, commercial or not-for-profit sector.

## Patient consent

Written informed consent for publication of their clinical details and clinical images was obtained from the patient.

## Author contribution statement

AAM collected data and composed the manuscript. TS is the physician responsible for the patient and has given permission to AAM to report this clinical case. Senior co-authors TS and JSN provided critical revision of the manuscript for important intellectual content.
